# Children's activities, parental concerns, and child care service utilization in the early stages of the COVID-19 pandemic

**DOI:** 10.3389/fpubh.2023.1047234

**Published:** 2023-06-29

**Authors:** Jasmine Zhang, Jackson Smith, Dillon Browne

**Affiliations:** ^1^Whole Family Lab, Department of Psychology, University of Waterloo, Waterloo, ON, Canada; ^2^Centre for Mental Health Research and Treatment, University of Waterloo, Waterloo, ON, Canada

**Keywords:** children's activities, child care services, COVID-19, latent profile analysis, parental concerns

## Abstract

**Introduction:**

In the early stages of the COVID-19 pandemic, most Canadian provinces and territories enacted public health measures to reduce virus spread, leading most child care centers across the country to limit or halt in-person service delivery. While it is broadly known that the range of activities available to children and youth reduced drastically as a result, research has yet to explore *if* and *how* children's activities shifted in relation to changes in child care arrangements.

**Method:**

Children's activities during the early months of the pandemic were assessed based on parent-report data (*n* = 19,959). Activity patterns were extracted via latent profile analysis. Thereafter, differences in child-care related outcomes across profiles were compared via logistic regression models.

**Results:**

Latent profile analysis yielded three distinct activity patterns: *Screenies* (91.5%) were children who engaged in high amounts of screen use relative to all other activities; *Analog* children (3.1%) exhibited mostly off-screen activities (e.g., reading, physical exercise); and children in the *Balanced* group (5.4%) appeared to pursue a wide variety of activities. Children were more likely to fall into the *Screenies* or *Balanced* profiles when caregivers reported changes in child care arrangements. Moreover, parents of children with *Balanced* activity profiles were more likely to be planning to use child care when services reopened post-pandemic, compared to parents of children in the *Analog* group.

**Discussion:**

The present findings call attention to heterogeneity in children's activities during COVID-19, which should be considered in the context of pandemic-related child care closures. Implications for children, families, and child care services during and beyond COVID-19 are discussed.

## 1. Introduction

Child care programs provide children with valuable experiences that foster socioemotional, cognitive, and academic growth (1). Unfortunately, this landscape shifted drastically due to service loss during the COVID-19 pandemic, which brought about extensive public health restrictions that significantly limited the range of activities available to children (2). A considerable body of literature has documented sedentary lifestyles in children during the pandemic comprising reduced physical activity and surges in digital media use (3, 4). However, there remains a need for more comprehensive examinations of children's activities during COVID-19 to better understand the wider impacts of reduced child care services on children's daily lives. The present study examined Canadian children's activity patterns and their associations with child care service utilization in the early months of COVID-19. This knowledge will provide insight on how the pandemic has affected children's opportunities to engage in developmentally enriching experiences, with important implications for post-pandemic planning in the child care sector.

When the World Health Organization declared the COVID-19 pandemic in March 2020 (5), governments across Canada implemented sweeping public health measures (e.g., physical distancing, working from home, and remote learning) to help limit virus spread. Although effective in reducing COVID-19 transmission (6), these restrictions created a myriad of disruptions that redefined normal life. Families with children have been particularly strained by the pandemic's downstream effects on social circumstances, with abrupt school and child care closures ranking among the most significant challenges (7–10). A nationwide survey of the Early Learning and Child Care Service sector in Canada reported that most child care centers and family care homes were closed between April 27 and May 1, 2020 (11). Attendance decreased dramatically in the centers that remained open; median enrolment fell from 50 children pre-COVID-19 to merely 5.5 children during the pandemic. These changes hold significant societal consequences. Child care constitutes a vital element of the circumstances in which children live, learn, and play; accordingly, 7, 2) maintain that “child care is a social determinant of health that crucially impacts the health, development, and economic wellbeing of children and families” [(8), p. 2]. Abundant literature suggests that child care participation facilitates cognitive development and socioemotional adjustment across the lifespan (12–14). Access to child care services also represents an important protective factor that fosters resilience within the family system, particularly for at-risk populations (15–17).

As the pandemic ensued, research from around the world documented striking declines in children's mental wellbeing (18, 19). Child care closures appear to underlie several mechanisms linked to these concerning trends, including increased unpredictability, disrupted routines, decreased in-person socialization, and reduced support from figures outside of the family home (20, 21). A related but currently understudied consequence is a marked reduction in the activities available to children in lockdown. Parents faced the daunting task of keeping their children safe while simultaneously offering activities that promote growth and learning. This was at the forefront of caregivers' minds in the early months of the pandemic. In a survey of caregivers in Pakistan during the pandemic, nearly three-quarters endorsed experiencing stress related to a lack of recreational opportunities for their children (22). Likewise, Lee et al. (10) reported that parents in the United States were most worried about the impacts of reduced physical activity, increased social isolation, and the loss of enriching experiences (e.g., extracurricular classes and religious services) on their children's wellbeing. Other studies highlighted increased sedentary screen use alongside reductions in physical activity as prominent sources of apprehension (23, 24). While concerning, these changes were inevitable (25, 26) and must be considered in tandem with the other activities in which children engaged. For example, Moore et al. (27) reported that Canadian children found creative ways to use their leisure time at home during lockdown, including arts and crafts, puzzles and games, and physical activities. Similarly, Stucke et al. (28) assessed U.S. and U.K. preschoolers' engagement in 32 activities during the initial months of the pandemic. Caregivers reported that children participated in a diverse set of activities, and playing with toys and physical games were ranked the most popular pastimes. Yet, beyond these findings, few studies have undertaken comprehensive examinations of children's activities aside from screen time and physical activity. Moreover, children's daily activities during COVID-19 were largely a result of child care service disruptions, but the links between these changes yet to be explored. Examining these associations will garner nuanced insight into the extent to which pandemic-related closures may have interacted with children's lifestyles.

From a service provision perspective, understanding children's activity patterns in the context of child care disruptions holds important implications for their social, recreational, and educational needs during and after the pandemic. Indeed, systemic formulations of the developmental ecology highlight that child care services encompass integral experiences that promote positive outcomes and the attainment of milestones (29, 30). For instance, the microsystem of Bronfenbrenner's (31) Ecological Systems Theory captures the immediate physical, financial, and social circumstances surrounding development. Children's activities are closely embedded within this level of development, as child care services represent a primary environment to access enriching pastimes and learning opportunities (29). Child care further creates a mesosystemic context for interactions between specific components of the microsystem (29). Examining the links between activity patterns and post-pandemic child care service utilization intentions could therefore lend additional knowledge to identifying children and families who are most in need of support.

The pandemic also raised many questions about the future of child care services (32, 33), many of which remain unanswered. Parents faced complex decisions about whether to enroll their children in care as they attempted to balance familial and occupational demands on the one hand and the risks of exposing their children to the virus on the other. Previous studies indicate that health and safety factors (e.g., gathering limits, exposure risk, and positive case counts) heavily influence parents' decisions on whether to send their children back to daycare and school following COVID-19 (34). However, other work indicates that caregivers also value the wide range of development-enhancing experiences offered by child care; quality-related factors such as access to activities for cognitive and social growth are among the most powerful drivers of parents' child care choices (35). This, combined with evidence supporting the benefits of child care services and parental concerns for children during lockdowns, suggests that decisions to access child care following COVID-19 could vary based on children's activities at home. Examining activity patterns thereby offers a unique and direct avenue to understanding the extent to which children's needs factor into service utilization following the pandemic. Knowledge of the factors related to parents' intentions about child care service utilization could help identify children and families who are most in need of support in the aftermath of COVID-19. This will further aid policymakers and service providers in developing supports for families with young children as the world adapts to the pandemic.

**Figure 1:**
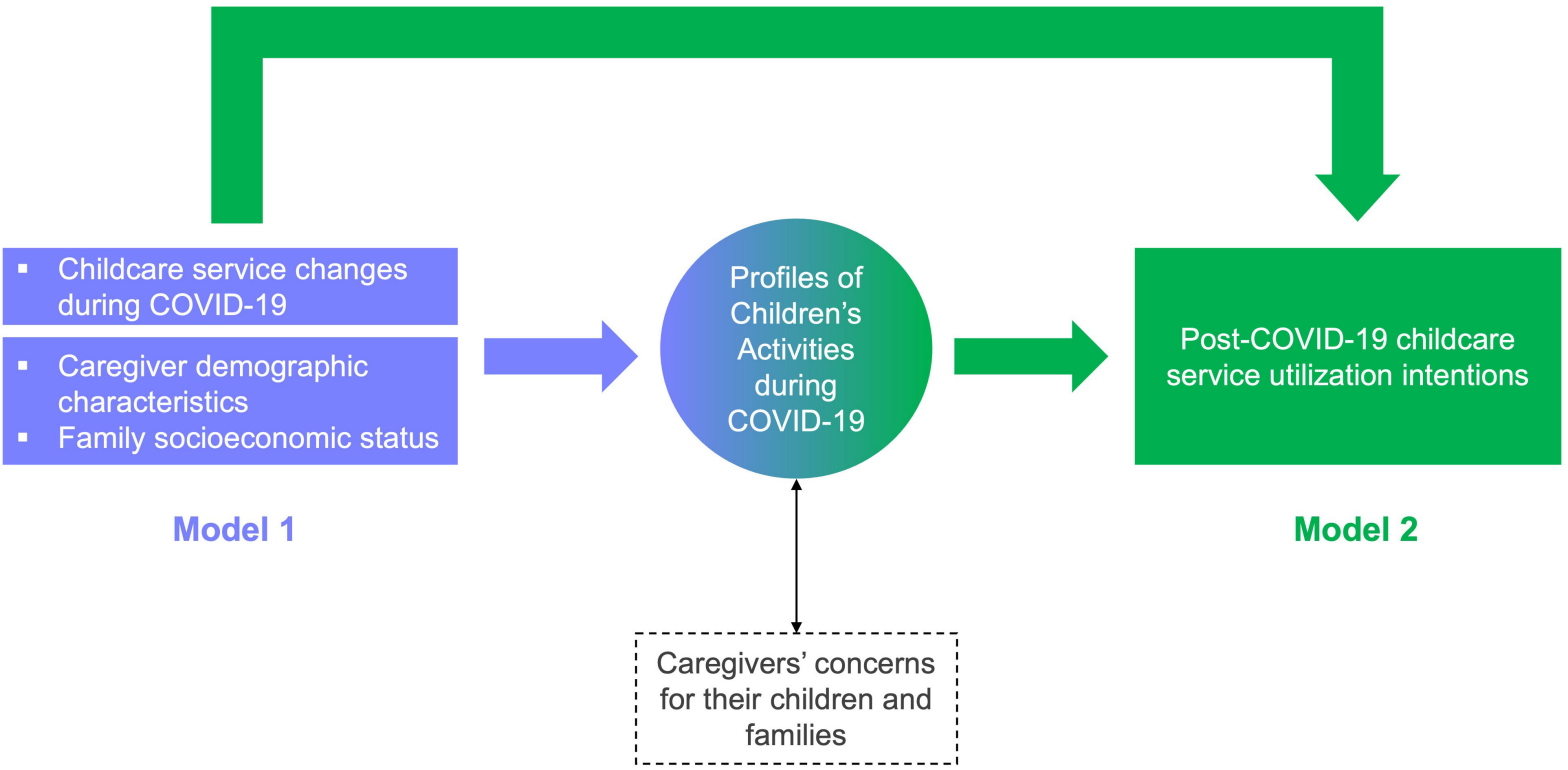
Conceptual model of the associations between study variables. We first established latent profiles of children's activities and validated these through comparing groups on the associated degree of parental concerns. Then, we evaluated a statistical model of the links between child care service changes during the COVID-19 pandemic and activity patterns, while considering sociodemographic characteristics (Model 1, components and paths depicted in blue). Thereafter, we examined links between children's activity patterns and their caregivers' post-pandemic child care service intentions, again accounting for sociodemographic characteristics (Model 2, components and paths depicted in green).

The present study sought to understand the links between children's activities, parental concerns, and child care service utilization in the early stages of COVID-19. The primary objectives were to explore patterns in children's activities (Objective 1) and assess their associations with parental concerns (Objective 2). We also aimed to examine child care service changes during the pandemic as predictors of children's activity patterns in the context of key sociodemographic characteristics (Objective 3). Finally, we evaluated the most salient factors, including children's activity patterns, linked to caregivers' child care utilization intentions for when services reopen (Objective 4). Figure 1 depicts a theoretical model that summarizes the study goals. By incorporating children's activity patterns, these findings will enhance the current understanding of Canadian families' child care needs in times of stress and unpredictability.

Given the exploratory nature of the methodology we employed to determine children's activity patterns (i.e., Latent Profile Analyses; LPA), we had several broad hypotheses. First, we predicted that distinguishable patterns (i.e., profiles) in children's activities would emerge, with some profiles characterized by high levels of sedentary behaviors (i.e., high screen use and low physical activity; Hypothesis 1). Given previous work illustrating that sedentary lifestyles were prominent sources of worry for caregivers during the pandemic, we predicted that these profiles would be linked with higher levels of parental concern (Hypothesis 2). While specific hypotheses were difficult to generate without prior knowledge about profiles, it is plausible that disruptions in child care may be associated with activity patterns that reflect higher levels of parental concern. We also hypothesized that children's activity patterns would vary in relation to whether they experienced changes in child care service utilization during the early months of COVID-19 (Hypothesis 3). Finally, we anticipated that caregivers' post-pandemic child care service utilization intentions would differ based on children's activity profiles (Hypothesis 4). Specifically, we expected caregivers to be more inclined to use services post-pandemic if their children's activity patterns were related to parental concerns and child care service changes (i.e., children forgoing enriching experiences at home). In contrast, caregivers whose children are engaged in developmentally appropriate and stimulating activities at home may be less intent on using services when they reopen.

## 2. Materials and methods

### 2.1. Participants and procedure

Data for this study were drawn from the *Impacts of COVID-19 on Canadians–Parenting During the Pandemic* (ICC-PDP) Data Collection Series (36, 37), which aimed to gather information regarding family functioning in the early stages of the pandemic. The data were collected by Statistics Canada, from a sample of Canadian caregivers (*N* = 32,228) with at least one child under 15 years old who resided in the same household. As outlined in the study documentation (37), the sample was crowdsourced, such that participants were self-selected through open advertising. Given this non-probabilistic approach to data collection, findings should not be generalized to draw conclusions about the larger population of Canadian adults who are caregivers to a child under 15 years old. The ICC-PDP dataset also includes a standardized benchmarking factor to correct for differing participation rates across three groups of families: those with children aged 0–5 years only, those with children aged 6–14 years only, and those with children aged 0–14 years (37). This benchmarking factor was applied as a weight in most statistical analyses.

Caregivers provided demographic information and reported on the pandemic's impacts on their child care service utilization, employment changes in the home, children's activities, and concerns for the wellbeing of their children and the overall family. In cases of families with multiple children, parents were instructed to provide an overall average. Caregivers were asked to consider the period from March 15, 2020 to the time of data collection, which took place from June 9–22, 2020. During this period, most regions in Canada enacted public health restrictions to mitigate virus spread. Lockdowns mandated the closure of non-essential businesses and restricted citizens from leaving their homes for non-essential reasons (38). Gatherings were prohibited in some provinces or restricted to small groups in others. With regard to education centers, most child care services were temporarily closed, and schools shifted to virtual learning (32, 36). As such, most participants in the present sample were likely experiencing some degree of COVID-19-related disruption at the time of data collection.

### 2.2. Measures

#### 2.2.1. Child care service utilization

##### 2.2.1.1. Changes in child care arrangements

The ICC-PDP survey included several items regarding child care service utilization. Caregivers answered the question, “*during the COVID-19 pandemic shutdown, have you used child care services for your child or children aged 0 to 14?*” Responses were coded in a binary manner (1 = *yes*, 0 = *no*). Subsequently, caregivers were prompted to further elaborate on their situation. Those who used child care services selected from the options of “*same child care arrangement and fees as pre-COVID-19*,” “*same child care arrangement but different fees (including no fees),”* “*different child care arrangement and fees (including no fees)*,” and “*different child care arrangement but same fees*.” Caregivers who did not use child care selected from the following options: “*did not attend child care and did not pay any fees*,” “*did not attend but paid child care fees to hold a space*,” and “*did not use child care prior to the COVID-19 pandemic*.”

**Figure 2:**
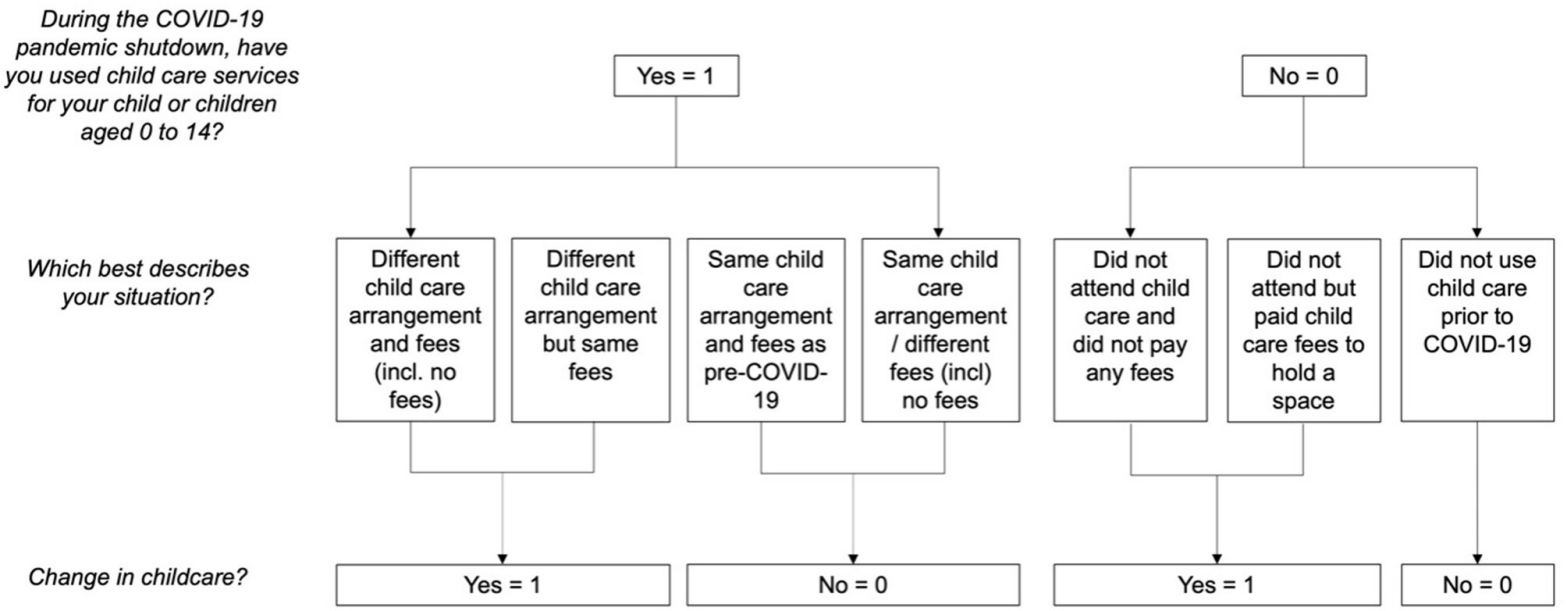
Flow chart depicting the response options and coding of child care service changes variables.

We created a binary variable to represent whether caregivers reported changes in child care based on their responses to the above questions (1 = *yes*, 0 = *no*). For parents who used services, responses of “*same child care arrangement and fees as pre-COVID-19*” or “*same child care arrangement but different fees (including no fees)”* were coded as not experiencing changes in child care on the new variable. Caregivers who selected “*different child care arrangement and fees (including no fees)*” or “*different child care arrangement but same fees*” were coded as “*yes*” to reflect experiencing changes in child care services. Participants who “*did not attend services and did not pay any fees”* or “*did not attend but paid child care fees to hold a space”* were also coded as “*yes”* to reflect experiencing a change in child care arrangements. Finally, caregivers who reported that they “*did not use child care prior to COVID-19*” were coded as not experiencing a change in child care services on the binary variable. Of note, these response options included information about changes in both child care arrangements and fees. We categorized participants based on the former, as this was more directly relevant to our study objectives. This means that whether participants experienced changes in child care fees did not influence coding. Figure 2 presents a visual flowchart illustrating the questions that were asked in the ICC-PDP survey, and the coding of these variables in the present study.

##### 2.2.1.2. Post-pandemic child care intentions

**Figure 3:**
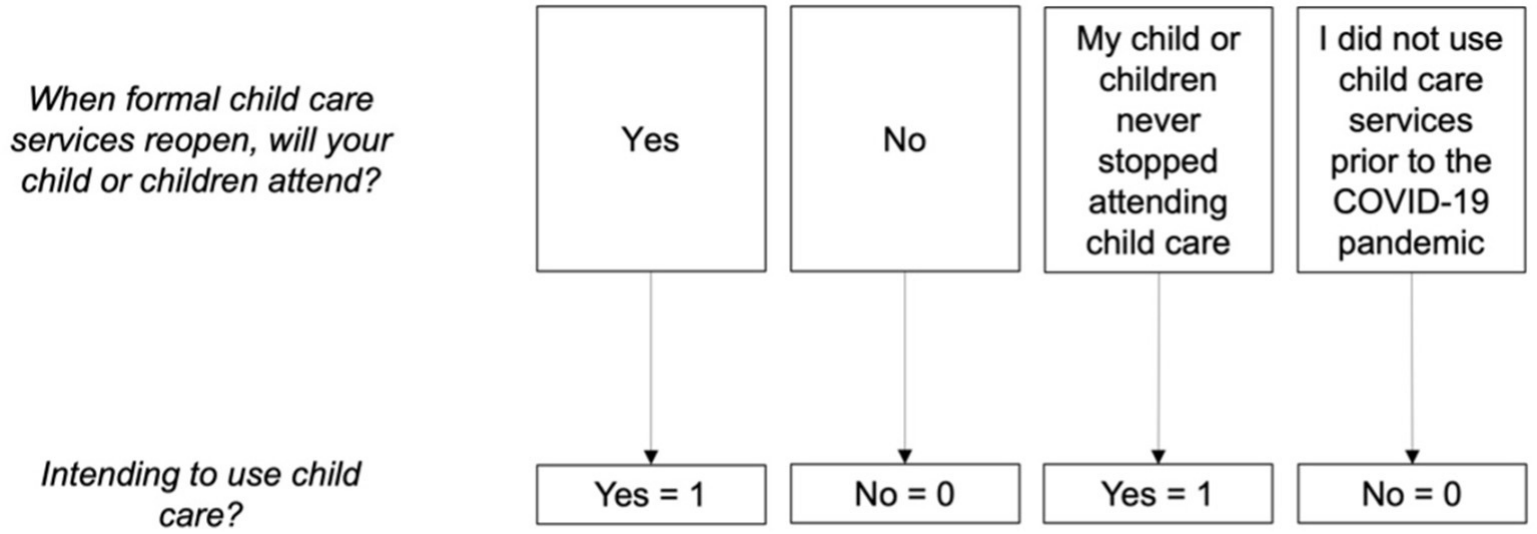
Flow chart depicting the response options and coding of post-COVID-19 child care service utilization intentions variables.

Caregivers' child care enrollment intentions for their children were assessed via the following item: “*when formal child care services reopen, will your child or children attend?*.” Response options included “*yes*,” “*no*,” “*my child or children never stopped attending child care*,” and “*I did not use child care services prior to the COVID-19 pandemic*.” The latter two options were recoded in a binary manner, such that “*my child or children never stopped attending child care*” was designated “*yes*,” and “*I did not use child care services prior to the COVID-19 pandemic”* was recoded as “*no*” (Figure 3).

#### 2.2.2. Children's activities at home during COVID-19

Caregivers reported on the extent to which their child or children engaged in various activities at home. Options included reading books or stories, using screen time (e.g., watching movies, videos, or television programs, playing games using any electronic device), playing games (e.g., cards, puzzles, board games), engaging in music, drama, or visual arts, doing physical activities (e.g., walking, cycling, dancing, yoga), participating in structured academic activities (e.g., worksheets, online school resources), and spending time developing other skills (e.g., cooking, sewing, gardening, crafts or making things). Frequency ratings were provided on a 4-point Likert scale, with points representing “*never, 1–2 times per week”*, “*3–5 times per week”*, and “*daily/almost every day”*. An additional option of “*not applicable*” was also included. However, these were recoded as missing values because they did not provide further information beyond the other options on the scale.

#### 2.2.3. Parental concerns

##### 2.2.3.1. Concerns for children

Caregivers responded to nine items that assessed the extent to which they held concerns about different aspects of their children's wellbeing during the COVID-19 pandemic. Areas probed included children's general physical health, general mental health, loneliness or isolation, school year and academic success, opportunities to socialize with friends, amount of screen time, online safety, amount of physical activity, and eating junk food or sweets. The level of concern for each item was rated on a 4-point Likert scale ranging from 1 = *not at all* to 4 = *extremely*. Although an additional option of “*not applicable*” was also included, this was recoded as missing data as it did not add further information.

##### 2.2.3.2. Concerns for the family

Parents' worries for the overall family unit during pandemic shutdowns were assessed via six items spanning the areas of staying connected with family or friends, getting along and supporting each other, balancing childcare, schooling and work, managing the child's or children's behaviors, stress levels, anxiety, and emotions, feeling lonely in the family home, and exhibiting negative behaviors toward the child or children (i.e., having less patience, using raised voices, scolding or yelling). Caregivers rated their level of concern for each item on a 4-point Likert scale (1 = *not at all* to 4 = *extremely*). An additional option of “*not applicable*” was also included. Again, these were recoded as missing responses.

#### 2.2.4. Covariates

##### 2.2.4.1. Caregiver age

Caregivers reported their age in years on the original ICC-PDP survey. In the public use microdata file available for download, parental age was provided as frequencies in the age brackets of 15–34 years, 35–44 years, 45–54 years, and 55+ years.

##### 2.2.4.2. Caregiver gender

In the original ICC-PDP questionnaire, caregivers were asked to report their gender through the options of “*male*,” “*female*,” or “*other*.” The publicly available dataset included imputed values, which were benchmarked based on sex (36). Hence, participants who originally reported their gender as *other* were randomly reassigned as either *male* or *female*. It is important to note that this imputation approach restricts the generalizability of the sample, and gender-related findings must therefore be interpreted with caution.

##### 2.2.4.3. Caregiver education

The ICC-PDP survey asked parents to provide the highest certificate, diploma, or degree that they completed. Options included “*less than high school diploma or its equivalent*,” “*high school diploma or a high school equivalency certificate*,” “*trades certificate or diploma*,” “*college, CEGEP or other non-university certificate or diploma*,” “*university certificate or diploma below the bachelor's level*,” “*Bachelor's degree*,” and “*university certificate, diploma or degree above the bachelor's level*.” However, in the ICC-PDP public use media file, caregiver education was only available as a binary variable representing whether caregivers attended university (1 = *yes*, 0 = *no*), which was included in statistical analyses.

##### 2.2.4.4. Employment status of family members

###### 2.2.4.4.1. Working from home

Caregivers completed several items addressing the impacts of the pandemic on the employment status of family members. Specifically, participants provided binary responses (1 = *yes*, 0 = *no*) regarding whether the following statements were true of their situation: “*someone in my family is working at a fixed location outside the home*,” “*someone in my family is working outside the home with no fixed location*,” and “*someone in my family is working from home*.” In the ICC-PDP public use microdata file, responses to these three items were collapsed into a single variable representing whether family members were working outside of the home or from home, with the following options: “*all family members working are doing so from home*,” “*all family members working are doing so outside the home,” and “mixed.”* These variables were recoded in a binary manner in the present study to reflect whether any family members were working from home (1 = *yes*, 0 = *no*). As such, responses of “*all family members working are doing so from home*” and “*mixed*” were recoded as “*yes*.” Responses of “*all family members working are doing so outside the home*” were recoded as “*no*.”

###### 2.2.4.4.2. Changes in employment status

COVID-19-related changes in work status were assessed via the following item: “*someone in my family lost their job, was laid off, or has reduced work hours due to COVID-19*.” Binary responses were provided (1 = *yes*, 0 = *no*).

### 2.3. Analytical plan

In our study's pre-registration (https://osf.io/3zb94/), we indicated that statistical analyses would only include data from parents whose children were not enrolled in child care services during the pandemic. This decision aimed to maximize responses from the group of caregivers who may be able to provide more accurate reports on their children's activities at home during the pandemic, which may have been difficult to report on if children were attending child care. However, this relies on the assumption that caregivers in the study sample would also be at home with their children. Upon reviewing the frequencies presented in the codebook, it was not evident that most caregivers were working from home in the presence of their children. Hence, we analyzed the full sample regardless of whether children attended child care services. This enabled wider coverage of caregivers who may have been under different circumstances, as well as the retention of more participants.

Data analysis proceeded in several stages. We conducted data cleaning and descriptive analyses using the *dplyr* (39) and *psych* (40) packages in RStudio. The benchmarking factor to correct for differing participation rates across three types of families was applied. Subsequently, we conducted an exploratory factor analysis (EFA) to determine the factor structure of parental concerns via the *REdaS* package (41). Data factorability was tested via the Kaiser-Meyer-Olkin (KMO) Measure of Sampling Adequacy and Barlett's Test of Sphericity, which examines the strength of the correlations across all the variables included in the factor analysis. KMO values of ≥ 0.50 and a statistically significant (*p* < 0.05). Bartlett's test statistic are desirable, as they suggest that factor analysis is an appropriate approach (42). The number of factors to be extracted was determined via a Scree plot and parallel analysis (43).

In the third stage of data analysis, we extracted patterns in children's activities via mixture modeling in Mplus Version 8.7. The seven child activities variables were subject to Latent Profile Analysis (LPA). Contrary to variable-centered approaches, which focus on examining relations among variables, person-centered techniques such as LPA aim to identify subgroups within a population based on a set of variables (44). We compared the solutions of models with two to seven profiles, then selected the best-fitting number of profiles based on Asparouhov and Muthen (44) recommendations. Specifically, we evaluated model fit through the Akaike information criterion (AIC), Bayesian information criterion (BIC), and the sample size adjusted Bayesian information criterion (aBIC) statistics. Low values on these statistics indicate stronger fit. We also relied on entropy statistics to determine the number of profiles. Entropy values can range from 0 to 1, where larger values indicate well-defined profiles with little ambiguity in group membership, and therefore, higher classification utility of the model. Furthermore, we conducted Lo-Mendell-Rubin (LMR) adjusted likelihood ratio tests to compare the fits of models with *k* profiles against the model with *k*-1 profiles.

Upon determining the number of profiles to extract, we assigned participants to the latent profile in which they had the highest probability of membership. Thereafter, we recoded resulting nominal profile membership into a series of dummy variables, with the group with the lowest levels of parental concerns as the reference. To validate latent profiles, we employed the BCH method in Mplus to compare the level of concerns held by caregivers in each latent class (45). This approach allows for tests of relationships between latent classes and an auxiliary outcome variable without causing shifts in latent class membership.

The final stage of analysis aimed to assess the associations between child care service utilization factors (i.e., changes during the pandemic and intentions following the pandemic), children's activity profiles, and parental concerns. We originally proposed path analysis to examine these relationships but shifted our analytical plan due to significant challenges with model fit. A logistic regression approach was adopted to better accommodate the categorical and binary nature of several study variables. We examined the predictive effect of child care service utilization changes on children's activity pattern profiles through a multinomial logistic regression model, conducted through the R package *nnet* (46). Predictors of profile membership included demographic characteristics (caregiver age, gender, education, and family employment changes during the pandemic), as well as child care service changes. Thereafter, we estimated a binary logistic regression model to examine the predictive relations between children's activity profile membership and caregivers' plans for child care once services re-open. Associations with demographic characteristics were also included in this model.

### 2.4. Missing data

Due to the non-probabilistic nature of the crowd-sourced sample, as well as the categorical nature of several items included in the study, we applied listwise deletion to remove data from participants who were missing values on any variable (47). The final sample included in statistical analyses consisted of *n* = 19,959 caregivers, which represents approximately two-thirds (61.93%) of the original ICC-PDP sample. The sociodemographic characteristics of both the initial study sample and the final sample included in analyses, reported in weighted frequencies and proportions, are displayed in Table 1.

**Table 1:** Frequencies of key demographic variables in the original study sample and the sample included in statistical analyses.

	**All participants (*****N*** = **32,228)**^**a**^		**Participants included in analyses (*****n*** = **19,959)**^**b**^	
	***n***	**%**	***n***	**%**
**Caregiver age**				
15–34 years	6,062	18.81	2,990	4.98
35–44 years	20,200	62.68	13,103	65.65
45–54 years	5,670	17.59	3,648	18.28
55+ years	296	0.92	81	0.41
**Caregiver gender** ^c^				
Female	29,060	90.17	18,014	90.26
Male	3,168	9.83	1,808	9.06
**Caregiver education**				
Did not attend university	8,213	25.49	4,798	24.04
Attended university	23,815	73.90	15,025	75.28
Missing	199	0.62	–	
**Province of residence**				
Newfoundland and Labrador	411	1.28	187	0.94
Prince Edward Island	120	0.37	64	0.32
Nova Scotia	742	2.30	445	2.23
New Brunswick	606	1.88	319	1.60
Quebec	7,238	22.46	4,175	20.92
Ontario	12,191	37.83	7,904	39.60
Manitoba	1,241	3.85	743	3.72
Saskatchewan	1,147	3.56	696	3.49
Alberta	4,408	13.68	2,794	14.00
British Columbia	3,989	12.38	2,434	12.20
Territories	134	0.42	61	0.31

Results are reported in weighted proportions, calculated using the benchmarking factor of family type based on child age. ^a^All participants refers to the original sample of caregivers included in the ICC-PDP study; ^b^Participants included in analyses refers to those who were retained following listwise deletion of cases with missing data; ^c^Parental gender was imputed for benchmarking based on sex, such that participants who reported a gender of “Other” were randomly reassigned a gender of either “Male” or “Female” (36).

## 3. Results

### 3.1. Descriptive statistics

**Figure 4:**
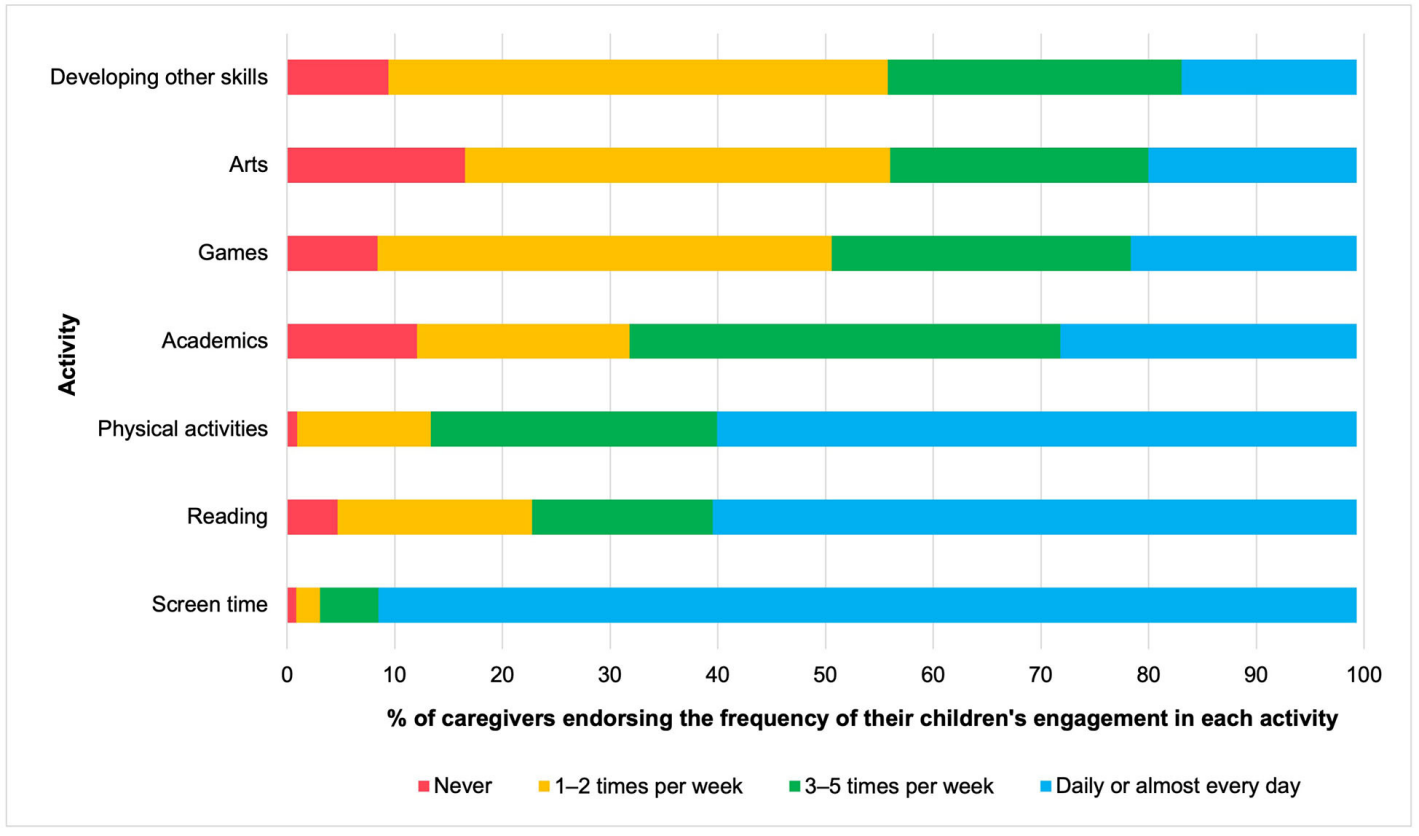
Children's engagement in activities by frequency, presented in proportions. Activities are ordered from least to most popular among children, based on the proportion of caregivers endorsing engagement in the activity *daily or almost every day*. Proportions are weighted based on the age range of children in the family. Reading, Reading books/stories; Arts, Music, drama, or visual arts; Academics, Structured academic activities; Other skills, Developing other skills.

Figure 4 presents the descriptive statistics of children's activities in weighted proportions, organized from the least to most frequently endorsed activities by caregivers. Children in the present sample engaged in all of the activities that were assessed in the ICC-PDP survey. However, the popularity of each activity varied. Most caregivers reported that their children read books or stories at least once per week (95.30%). A very small proportion reported that their children never used screen-based devices, while the rest noted that their children engaged in screen time daily or almost every day (99.17%). Almost all (98.39%) caregivers reported that their children participated in physical activity at least once per week, and a majority also reported that their children spent time on structured academic activities (87.94%), playing games (i.e., cards, puzzles, board games; 91.63%), doing creative activities (i.e., music, drama, or visual arts; 83.50%), and developing other skills (90.59%).

**Figure 5:**
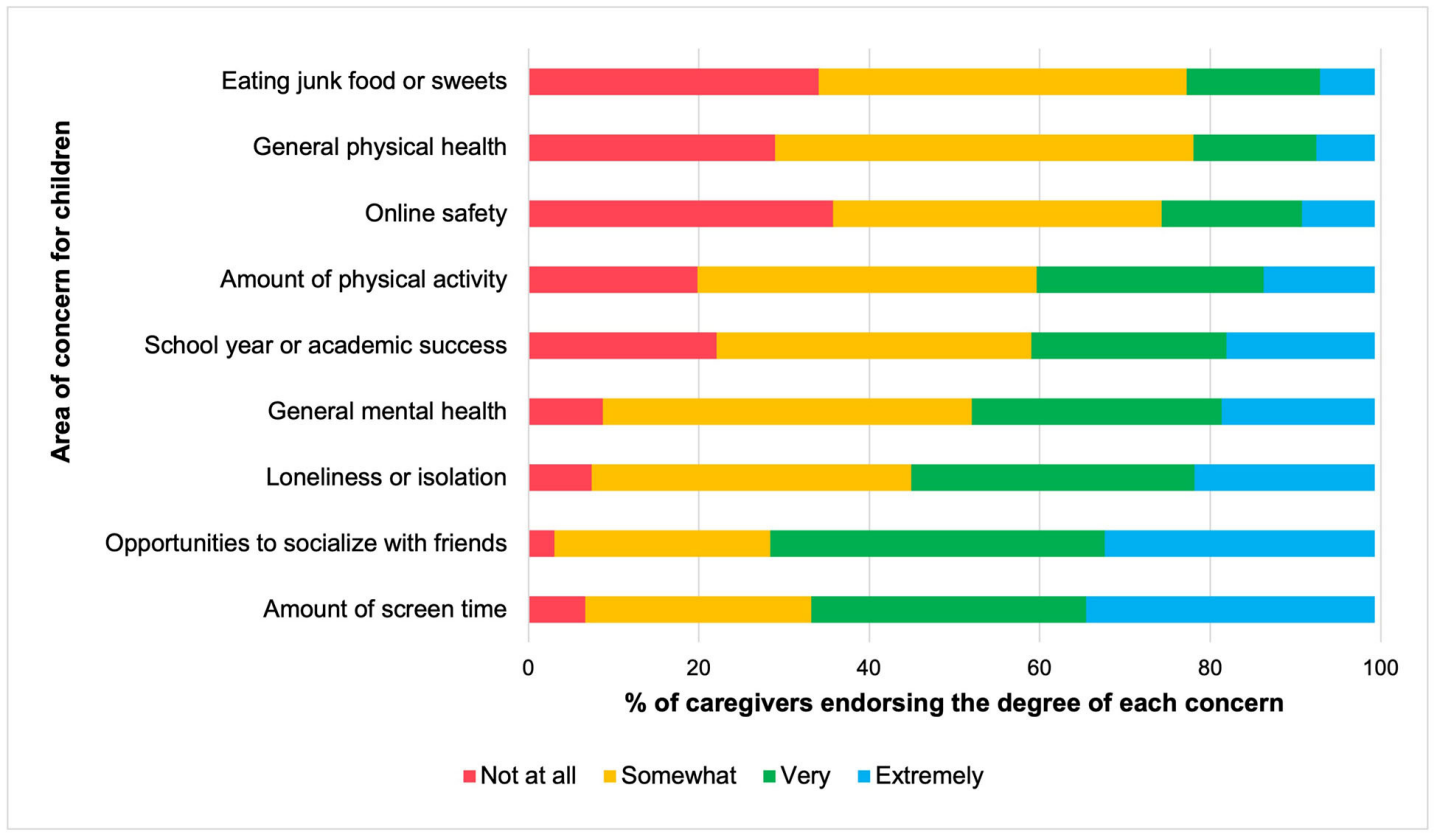
Parental concerns for children by frequency, presented in proportions. Concerns are ordered from least to most frequently endorsed among caregivers, based on the proportion who endorsed being *extremely* concerned about each area. Proportions are weighted based on the age range of children in the family.

**Figure 6:**
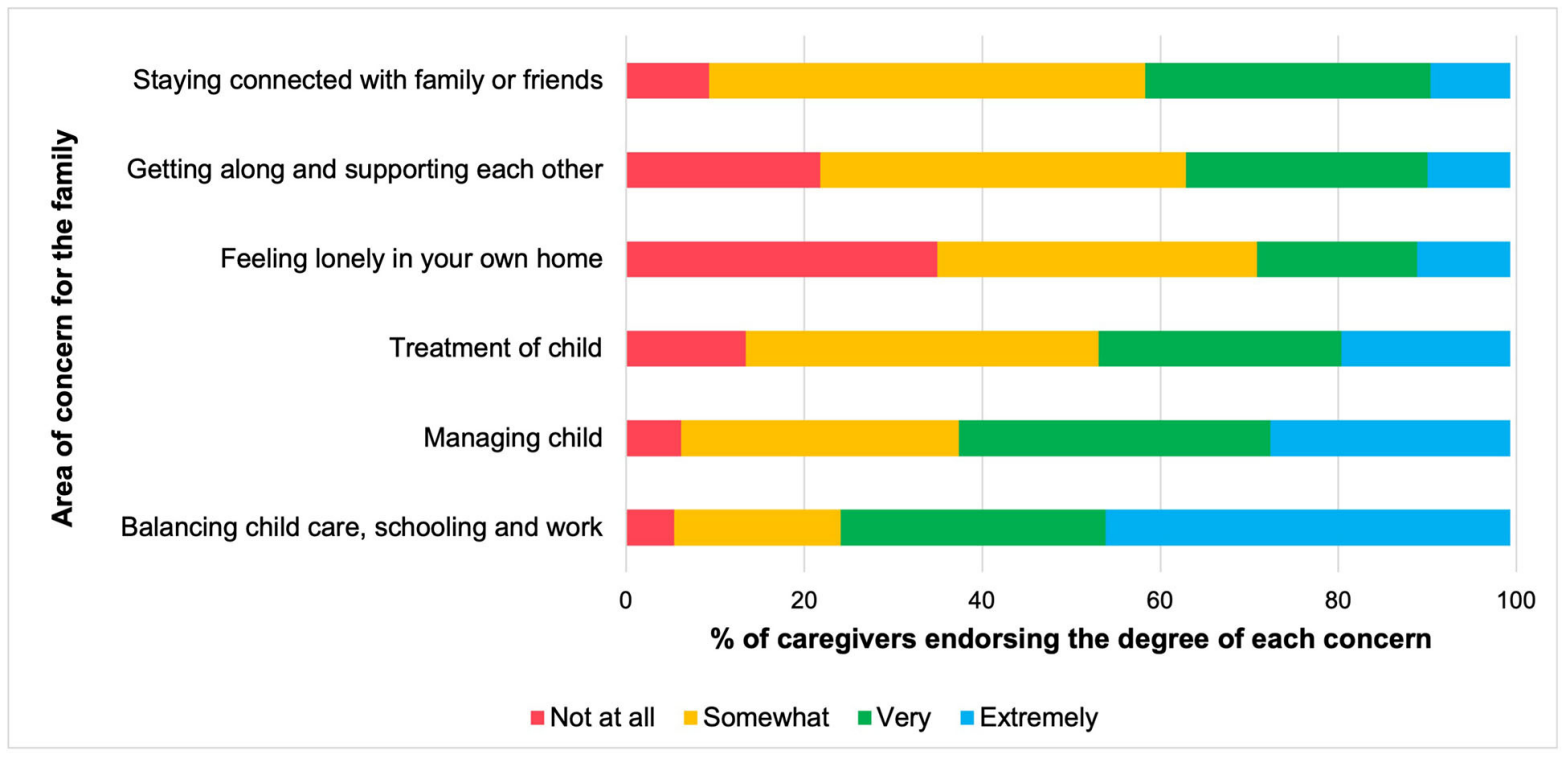
Parental concerns for the family by frequency, presented in proportions. Concerns are ordered from least to most frequently endorsed among caregivers, based on the proportion who endorsed being extremely concerned about each area. Proportions are weighted based on the age range of children in the family. Managing child, Managing your child's or children's behaviors, stress levels, anxiety, emotions; Treatment of child, Having less patience, raising your voice, scolding or yelling at your child or children.

Caregivers also endorsed various areas of concern for their children and families during the pandemic shutdown. Figure 5 depicts concerns from least to most highly endorsed. Moderate levels of worry regarding children's general mental and physical wellbeing emerged. Most participants (92.57%) reported being at least “somewhat” concerned about their children being lonely or isolated; worries about reduced socialization opportunities were also prevalent (96.27% expressed being somewhat, very, or extremely concerned). Furthermore, most parents (93.33%) reported some degree of concern about the amount of screen time that their children were engaging in. In terms of family-related concerns (Figure 6), caregivers reported the highest degrees of worry about the ability to balance child care, schooling, and work (94.58% endorsed being somewhat, very, or extremely concerned). Relatedly, the family's ability to manage their children's behaviors was a prominent area of concern.

### 3.2. Exploratory factor analysis of parental concerns

Initially, we examined the factorability of the 15 parental concerns items. As shown in Table 2, which displays Spearman inter-item correlations between study variables, all concern items were significantly and positively correlated. Tests of assumptions revealed that factor analysis was appropriate: the KMO Measure of Sampling Adequacy was 0.89, and Bartlett's Test of Sphericity was statically significant [χ^2^(105) = 101,372.63, *p* < 0.001]. These results indicated that the strength of partial correlations between the parental concern variables was adequate, and therefore supported the use of a factor-analytic approach. Examinations of a Scree plot and parallel analysis indicated a one-factor solution. Table 3 displays the loadings of each item onto a latent concerns construct. These results suggested that parental concerns about their child and family would be best represented as one construct in the present study. Thus, we conducted all further analyses using an overall concerns variable derived from calculating the mean of each participant's responses across all items that assessed concerns for children and the overall family.

**Table 2:** Bivariate Spearman correlations of study variables.

	**1**	**2**	**3**	**4**	**5**	**6**	**7**	**8**	**9**	**10**	**11**	**12**	**13**	**14**	**15**	**16**	**17**	**18**	**19**	**20**	**21**
**Child activities**																					
1. Reading																					
2. ST	−0.09^**^																				
3. Games	0.27^**^	0.01																			
4. Arts	0.29^**^	−0.06^**^	0.31^**^																		
5. Phy act	0.34^**^	−0.06^**^	0.21^**^	0.24^**^																	
6. Academ	0.01	0.14^**^	0.04^**^	0.00	0.01																
7. Other	0.25^**^	−0.06^**^	0.25^**^	0.34^**^	0.29^**^	0.07^**^															
**Concerns for children**																					
8. Phys hlth	−0.10^**^	0.05^**^	−0.02^**^	−0.05^**^	−0.19^**^	0.01	−0.07^**^														
9. Ment hlth	−0.09^**^	0.12^**^	−0.02^**^	−0.07^**^	−0.12^**^	0.01^*^	−0.06^**^	0.45^**^													
10. Lonely	−0.05^**^	0.10^**^	−0.02^*^	−0.05^**^	−0.10^**^	−0.03^**^	−0.06^**^	0.32^**^	0.67^**^												
11. School success	−0.22^**^	0.14^**^	−0.08^**^	−0.15^**^	−0.15^**^	0.17^**^	−0.12^**^	0.23^**^	0.36^**^	0.31^**^											
12. Social	0.02^*^	0.07^**^	0.01	−0.04^**^	−0.05^**^	−0.05^**^	−0.04^**^	0.17^**^	0.46^**^	0.59^**^	0.31^**^										
13. Amt ST	−0.20^**^	0.32^**^	−0.12^**^	−0.19^**^	−0.21^**^	0.04^**^	−0.20^**^	0.22^**^	0.32^**^	0.29^**^	0.33^**^	0.26^**^									
14. Online safety	−0.22^**^	0.15^**^	−0.08^**^	−0.12^**^	−0.18^**^	0.22^**^	−0.08^**^	0.27^**^	0.30^**^	0.22^**^	0.35^**^	0.12^**^	0.40^**^								
15. Amt phys act	−0.20^**^	0.16^**^	−0.11^**^	−0.16^**^	−0.38^**^	0.08^**^	−0.19^**^	0.44^**^	0.39^**^	0.35^**^	0.34^**^	0.29^**^	0.44^**^	0.40^**^							
16. Junk food	−0.24^**^	0.17^**^	−0.09^**^	−0.13^**^	−0.24^**^	−0.00	−0.13^**^	0.27^**^	0.29^**^	0.26^**^	0.29^**^	0.18^**^	0.41^**^	0.36^**^	0.47^**^						
**Concerns for the family**																					
17. Stay connected	0.03^**^	−0.01	0.04^**^	0.00	−0.00	−0.05^**^	0.00	0.22^**^	0.34^**^	0.36^**^	0.20^**^	0.38^**^	0.15^**^	0.16^**^	0.24^**^	0.17^**^					
18. Get along	−0.03^**^	0.04^**^	0.00	−0.04^**^	−0.05^**^	−0.03^**^	−0.03^**^	0.26^**^	0.39^**^	0.34^**^	0.24^**^	0.27^**^	0.23^**^	0.23^**^	0.27^**^	0.24^**^	0.50^**^				
19. Balance	0.06^**^	0.07^**^	0.02^**^	−0.02^**^	0.01	−0.04^**^	−0.01	0.13^**^	0.28^**^	0.27^**^	0.24^**^	0.29^**^	0.26^**^	0.13^**^	0.19^**^	0.16^**^	0.27^**^	0.34^**^			
20. Manage child	−0.04^**^	0.13^**^	−0.01	−0.05^**^	−0.08^**^	−0.01	−0.05^**^	0.27^**^	0.58^**^	0.51^**^	0.32^**^	0.40^**^	0.33^**^	0.26^**^	0.33^**^	0.29^**^	0.36^**^	0.50^**^	0.46^**^		
21. Lonely at home	−0.08^**^	0.03^**^	−0.01	−0.06^**^	−0.10^**^	−0.10^**^	−0.07^**^	0.20^**^	0.35^**^	0.39^**^	0.21^**^	0.30^**^	0.22^**^	0.17^**^	0.24^**^	0.24^**^	0.33^**^	0.41^**^	0.21^**^	0.39^**^	
22. Child treatment	0.04^**^	0.08^**^	0.00	−0.06^**^	−0.02^*^	−0.10^**^	−0.07^**^	0.14^**^	0.33^**^	0.30^**^	0.20^**^	0.28^**^	0.28^**^	0.11^**^	0.19^**^	0.25^**^	0.25^**^	0.43^**^	0.38^**^	0.51^**^	0.42^**^

^*^p < 0.05. ^**^p < 0.01. Weights were not applied in correlations. Reading, Reading books/stories; ST, Screen time; Arts, Music, drama, or visual arts; Phy act, Physical activities; Academ, Structured academic activities; Other skills, Developing other skills; Phy hlth, General physical health; Ment hlth, General mental health; Lonely, Loneliness or isolation; School success, Socializing, Opportunities to socialize with friends; Amt. screen time, Amount of screen time; Junk food, Eating junk food or sweets; Stay connected, Staying connected with family or friends; Get along, Getting along and supporting each other; Balance, Balancing child care, schooling and work; Manage child, Managing your child's or children's behaviors, stress levels, anxiety, emotions; Lonely at home, Feeling lonely in your own home; Child treatment, Having less patience, raising your voice, scolding or yelling at your child or children.

**Table 3:** Factor loadings of parental concerns indicators onto an overall concerns latent variable.

**Item**	**Loading**
**Concerns for children**	
General physical health	0.46
General mental health	0.76
Loneliness or isolation	0.72
School year and academic success	0.48
Opportunities to socialize with friends	0.59
Amount of screen time	0.49
Online safety	0.41
Amount of physical activity	0.55
Eating junk food or sweets	0.46
**Concerns for the family**	
Staying connected with family or friends	0.51
Getting along and supporting each other	0.59
Balancing child care, schooling and work	0.47
Managing child	0.74
Feeling lonely in your own home	0.53
Treatment of child	0.53

Managing child, Managing your child's or children's behaviors, stress levels, anxiety, emotions; Treatment of child, Having less patience, raising your voice, scolding or yelling at your child or children.

### 3.3. Children's time use profiles and parental concerns

Model fit statistics of children's latent activity profiles are presented in Table 4. Entropy values were similar across all models, ranging from 0.82 to 1.00. The AIC, BIC, and aBIC values decreased from models with one through six profiles, then began to increase in the seven-profile solution. Although this may suggest that a six-profile solution represents the best fit to the data, model estimation was unreliable for models with over five profiles. A solution comprising six profiles was also difficult to interpret due to the presence of many profiles with few clear differences in activity patterns between them. Furthermore, LMR adjusted likelihood ratio tests suggested that two- and three-profile solutions fit the data significantly better than solutions with *k*−1 profiles. This test was not significant for solutions with four or more profiles. A three-class solution was therefore deemed the best-fitting model. Random starts and final stage optimizations for the three-profile solution were increased, to which the optimal log-likelihood was robust. Examination of the distribution of participants across profiles indicated that each group included a sizable number of members (i.e., all three profiles contained over 600 participants).

**Table 4:** Fit indices for latent profile models of child activities.

**Number of profiles**	**AIC**	**BIC**	**aBIC**	**Entropy**	**pLMR**
2	316255.97	316429.80	316359.88	1.00	<0.001
**3**	**281840.62**	**282077.66**	**281982.33**	**1.00**	**0.032**
4	267822.21	268122.46	268001.70	0.95	0.138
5	264851.20	265214.67	265068.48	0.82	0.410
6^a^	258783.26	259209.94	259038.33	0.89	–
7^b^	261904.64	262394.52	262197.49	0.83	–

A three-class solution (in bold) was evaluated to be the best-fitting model. AIC, Akaike information criterion; BIC, Bayesian information criterion; aBIC, sample size adjusted Bayesian information criterion; p (LMR), p-value of the LMR adjusted likelihood ratio test for k versus k-1 classes. ^a, b^The best loglikelihood was not replicated in the six- and seven-profile solutions, and was reported to be untrustworthy due to local maxima. The likelihood ratio test for these models could not be computed due to model non-convergence.

**Figure 7:**
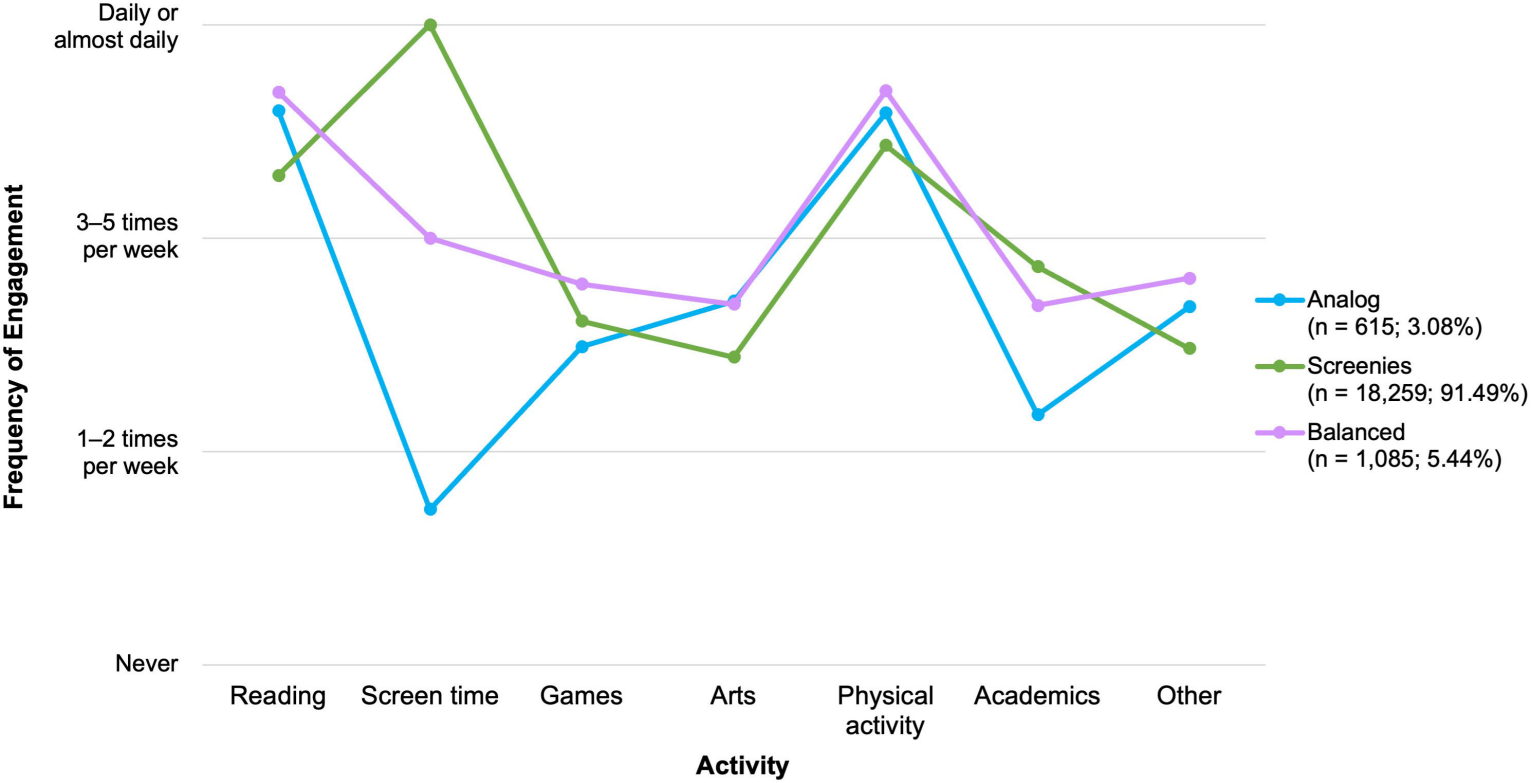
Latent profiles of children's activities. Reading, Reading books/stories; Arts, Music, drama, or visual arts; Academic, Structured academic activities; Other skills, Developing other skills.

Figure 7 displays the final three-profile solution representing children's latent activity profiles. Children in the largest group, the *Screenies* (*n* = 18,259; 91.49%), engaged in more screen time relative to all other activities. A second profile (*n* = 1,085; 5.44%) included children who seemed to be engaging in a wider variety of activities. Children with these *Balanced* profiles read books or stories and engaged in physical exercise on a near-daily basis. They also used screens, participated in structured academic activities, and spent time developing other skills several times per week. Finally, a small group of children (*n* = 615; 3.08%) appeared to read and do physical exercise on a near-daily basis while using relatively little screen time. These children also engaged in lower levels of structured academic activities. As such, they were designated the *Analog* group to reflect a lifestyle that was more off-screen in nature.

Caregivers of children with each activity profile (*Analog, Screenies*, and *Balanced*) reported slight variations in their overall level of concern for children and families. Specifically, parents of children in the *Analog* profile reported the lowest levels of concern with a mean of 2.0 (*SE* = 0.03), representing being “somewhat” worried. Parents of *Screenies* noted slightly higher mean concerns (*M* = 2.5, *SE* = 0.01), which descriptively reflects being between “somewhat” and “very” concerned. Finally, parents of children who were classified in the *Balanced* group reported a mean concern level of 2.2 (*SE* = 0.02). This most closely corresponds to being “somewhat” concerned. To further explore these differences, we conducted equality tests of concern levels across profiles (*Analog, Screenies*, and *Balanced*) via the BCH method in Mplus. The overall test was significant (χ^2^ = 512.45, *p* < 0.001). Results also revealed significant differences between all three groups (Table 5). Caregivers of children in the *Analog* group tended to rate significantly lower levels of concerns compared to parents of children in the *Screenies* (χ^2^ = 316.99, *p* < 0.001) and *Balanced* profiles (χ^2^ = 38.63, *p* < 0.001). Parents of the *Screenies* group reported significantly higher concerns compared to children from the *Balanced* group (χ^2^ = 219.12, *p* < 0.001). Of note, the interpretation of these results must be qualified by both the small magnitude of the between-group differences, as well as the study's large sample size. As such, these group differences in parental concerns likely hold limited practical implications.

**Table 5:** Mean parent concerns and pairwise comparisons across profiles.

	**General concern**				
**Latent profile**	***M***	**(** ***SE*** **)**		χ^2^	***p*** **-value**
*Analog*	2.0	(0.03)	*Analog* vs. *Screenies*	769.71	<0.001
*Screenies*	2.5	(0.01)	*Analog* vs. *Balanced*	405.39	<0.001
*Balanced*	2.2	(0.02)	*Screenies* vs. *Balanced*	239.03	<0.001

### 3.4. Multinomial logistic regression model predicting children's activity profiles

Following the estimation of latent profiles to represent activity patterns, we performed multinomial logistic regression analyses to examine the extent to which changes in child care service arrangements and family demographic covariates predicted children's latent activity group membership (Model 1). The *Analog* profile was designated as the reference due to having the lowest level of parental concerns, making it a more neutral group for comparisons. A likelihood ratio test revealed significant increases in model fit with the addition of predictor variables, as compared with a null model containing only the intercept [χ^2^(12) = 373.50, *p* < 0.001].

#### 3.4.1. Predictors of balanced vs. analog groups

Table 6 displays the full results of Model 1. The first set of comparisons aimed to establish the predictors of whether children were assigned to the *Balanced* group vs. the *Analog* group. Odds ratios (ORs) revealed that when caregivers reported experiencing changes in child care services, their children were 1.53 times more likely to exhibit a *Balanced* activity profile than an *Analog* profile (*B* = 0.43, *p* < 0.001). All demographic predictors of membership in the *Balanced* group were significant. Specifically, each increase in caregivers' age group was related to a 1.57 times higher likelihood of being assigned to the *Balanced* group (*B* = 0.45, *p* < 0.001). However, children were 30% less likely to be assigned to the *Balanced* time use profile when their caregivers were male (OR = 0.70; *B* = −0.36, *p* = 0.027). Children of caregivers who reported attending university were also less likely to be classified in the *Balanced* activity profile compared to the *Analog* profile (OR = 0.64; *B* = −0.44, *p* = 0.001). Regarding COVID-19 employment changes, whether family members worked from home or experienced job loss (i.e., lost their job, were laid off, or had reduced work hours) due to the pandemic did not significantly predict children's membership in the *Balanced* vs. *Analog* groups.

**Table 6:** Multinomial logistic regression model examining predictors of latent activity profile membership (Model 1).

	**Balanced vs. Analog**				**Screenies vs. Analog**			
	***B***	***(SE)***	**OR**	**OR CI_0.95_**	***B***	***(SE)***	**OR**	**OR CI_0.95_**
**Effect**								
Intercept	−0.18	(0.21)	0.84	0.55–1.27	1.69^**^	(0.17)	5.40	3.86–7.56
Child care change	0.43^**^	(0.11)	1.53	1.25–1.89	0.38^**^	(0.09)	1.45	1.23–1.72
**Caregiver demographics**								
Age	0.45^**^	(0.09)	1.57	1.31–1.88	1.08^**^	(0.07)	2.94	2.54–3.41
Gender	−0.36^*^	(0.16)	0.70	0.51–0.96	−0.55^**^	(0.13)	0.58	0.45– 0.74
University attendance	−0.44^**^	(0.14)	0.64	0.49–0.84	−0.71^**^	(0.11)	0.49	0.39–0.62
**Family employment**								
Working from home	0.09	(0.14)	1.09	0.84–1.42	0.12	(0.11)	1.13	0.91–1.40
Job loss	0.02	(0.11)	1.02	0.82– 1.26	−0.10	(0.09)	0.90	0.76–1.07

^*^p < 0.05, ^**^p < 0.01.

#### 3.4.2. Predictors of screenies vs. analog groups

The second set of comparisons in Model 1 examined the predictors of being assigned to the *Screenies* group vs. the *Analog* group. A significant intercept suggested that children were approximately five times more likely to be assigned to the *Screenies* group (OR = 5.40; *B* = 1.69, *p* < 0.001) before adding predictors to the model. Experiencing changes in child care attendance significantly increased children's likelihood of being a *Screenie* by 45% (OR = 1.45; *B* = 0.38, *p* < 0.001), as did having an older parent (OR = 2.94; *B* = 1.08, *p* < 0.001). However, having caregivers who were male (OR = 0.58; *B* = −0.55, *p* < 0.001) or who attended university (OR = 0.49; *B* = −0.71, *p* < 0.001) were both associated with lower odds of being assigned to the *Screenies* group than the *Analog* group. Again, having family members work from home or experience job loss during COVID-19 did not predict membership in the *Screenies* profile over the *Analog* profile.

### 3.5. Binary logistic regression model predicting post-pandemic child care service utilization intentions

The second set of analyses aimed to identify the factors that were associated with parents' intentions to enroll their children in child care when services reopened. We estimated a binary logistic regression model (Model 2), presented in Table 7, to examine children's activity profiles (*Balanced* vs. *Analog, Screenies* vs. *Analog*) and family demographic characteristics as predictors of caregivers' child care plans. Model 2 showed statistically significant improvements in fit compared to a null model that did not include predictors (Table 7) [χ^2^(7) = 1123.36, *p* < 0.001].

**Table 7:** Binary logistic regression model examining predictors of post-pandemic childcare service attendance intentions (Model 2).

**Effect**	**Estimate**	***(SE)***	**OR**	**OR CI_0.95_**
Intercept	0.46^**^	(0.10)	1.58	1.29–1.94
**Activity profile**				
*Screenies* vs *Analog*	0.05	(0.09)	1.05	0.88–1.25
*Balanced* vs *Analog*	0.21^*^	(0.11)	1.24	1.00–1.53
**Caregiver demographics**				
Age	−0.84^**^	(0.03)	0.43	0.41–0.46
Gender ^a^	0.31^**^	(0.05)	1.37	1.24–1.52
University attendance	0.50^**^	(0.04)	1.66	1.53–1.79
**Family employment**				
Working from home	0.12^**^	(0.04)	1.13	1.04–1.23
Job loss	−0.17^**^	(0.03)	0.85	0.79–0.90

^*^p < 0.05, ^**^p < 0.01; ^a^Female gender was designated the reference group.

A significant intercept (OR = 1.58, *B* = 0.46, *p* < 0.001) in Model 2 suggested that parents were typically more likely to plan to have their children attend child care services when they reopened, before including other predictors. In terms of children's activity profiles, caregivers of *Screenies* did not report significantly different child care service utilization intentions compared to parents of *Analog* children. Interestingly, parents of children with *Balanced* activity patterns were more likely to report planning to send their children to child care when services reopened compared to parents of *Analog* children (OR = 1.24; *B* = 0.21, *p* = 0.046). Caregivers who were male (OR = 1.37, *p* < 0.001) and attended university (OR = 1.66, *p* < 0.001) were also more likely to plan for their children to attend child care services upon reopening. In contrast, higher caregiver age was related to a lower likelihood of future service use (OR = 0.43; *B* = −0.84, *p* < 0.001). Regarding employment, caregivers were slightly more likely to report planning to have their children attend child care if at least one family member was working from home (OR = 1.13; *B* = 0.12, *p* < 0.005). Experiencing employment loss due to the pandemic was associated with a reduced likelihood of utilizing child care services (OR = 0.84, *p* < 0.001).

## 4. Discussion

The present study aimed to delineate associations between Canadian children's activities, parental concerns, and child care utilization during the COVID-19 pandemic. Analyses revealed that children's participation in various pastimes combined to create three meaningful profiles, which we named *Screenies, Analog*, and *Balanced*. These patterns were associated with parental concerns, which were highest for the *Screenies* group, followed by the *Balanced* and *Analog* groups. Profile membership also interacted with aspects of pandemic-related child care service utilization. Experiencing changes in child care arrangements in March–June 2020 predicted a higher likelihood of membership in the *Balanced* and *Screenies* groups over the *Analog* group. Additionally, parents of children in the *Balanced* group were more likely to endorse intentions to use child care services following the pandemic compared to parents of *Analog* children. These findings collectively illustrate heterogeneity in how children and families responded to child care disruptions during COVID-19, with important implications for post-pandemic planning.

### 4.1. Children's activity profiles during the pandemic

Reflecting the reality that increased digital media use was inevitable during the pandemic, an overwhelming majority of children in the present study were designated *Screenies*. This group of children engaged in daily screen use and some physical exercise but participated less in other activities. This pattern converges with a considerable amount of literature to suggest that most children were highly reliant on screen-based devices to access social, educational, and recreational opportunities during COVID-19 (48, 49). Notably, not all children in the present study exhibited activity patterns dominated by screen use. A small group exhibited an *Analog* activity profile that comprised more non-digital activities (e.g., reading books and stories, games) and lower amounts of screen time relative to the other profiles. As such, *Analog* children may represent those who adjusted to the pandemic by turning to offline activities. Several alternative explanations should also be considered. Past studies consistently show that screen time increases across childhood and adolescence (50, 51) and the *Analog* group may have comprised younger children who naturally engaged in less digital media use. Moreover, inconsistent access to digital technologies (e.g., internet, devices) increased the vulnerability of many Canadian children by reducing opportunities to participate in virtual activities (52). This warrants future research on the specific mechanisms that relate to different levels of on- vs. off-screen activities both during and after the pandemic, particularly as activity profiles were most divergent in their screen use in the present study. Finally, children who exhibited a *Balanced* profile seemed to engage in the widest variety of activities. This group showed moderate levels of physical exercise and screen use, and pursued various other endeavors (e.g., creative arts, and developing other skills). As access to diverse activities in childhood is central to positive outcomes (53), a *Balanced* activity profile may be linked to benefits across multiple domains of development, particularly during COVID-19. However, it is also essential to examine the extent to which this may be feasible in unique circumstances such as the pandemic. Future work should assess activity patterns as a principal social determinant of wellbeing and its role within specific contexts in which activities are less accessible. Overall, variations in children's activity patterns detected in the present study illustrate that children's responses to the pandemic likely differ. Post-pandemic efforts to promote wellbeing and foster healthy lifestyles in children should be designed with the needs of specific groups of children in mind.

### 4.2. Children's activity patterns and parental concerns

The present study found small but significant variations in parental worries across children's activity profiles. Consistent with our second hypothesis, parents of *Screenies* expressed the highest levels of concern, followed by the *Balanced*, then *Analog* groups. The largest between-group differences emerged in children's screen use frequency, suggesting that this activity may have been a primary contributor to parents' worries. This aligns with considerable literature documenting excessive screen time as a top-priority concern that parents felt for children during lockdowns (23, 24). Nevertheless, only a small correlation between overall parental concerns and children's screen use frequency emerged in the present study. Screen use constituted a major aspect of altered lifestyles during the pandemic, and though this became the primary activity for many children in the present study, a subset seems to have incorporated digital media as one of several similarly pursued pastimes. A more nuanced interpretation could therefore suggest that parents' concerns are more closely associated with children's screen use levels *relative to* their engagement in other activities, rather than the absolute amount of screen time in and of itself. Consequently, digital media use may be a notable but not standalone indicator of children's activities—nor is it an independent target of caregiver concern during the pandemic. Strategies to address parents' worries for their children's wellbeing could take this into consideration by promoting engagement in a wide range of activities, rather than merely promoting reductions in screen use (54). Notwithstanding, it is also possible that the correlation between children's screen use and parents' concerns in the present study was, in part, due to the use of a one-dimensional measure (i.e., average score) across specific indicators of concerns. Of these, only one indicator captured worries about screen use, with a moderate factor loading. It is further important to note that between-group differences in concern levels were quite small and the large sample size may have inflated the statistical significance of these differences. Ongoing work must continue exploring links with parental perceptions and concerns to further validate profiles.

### 4.3. Activity profiles and child care service utilization

Examining associations between children's activities and child care service utilization in the present study provided additional insight into the ways in which the pandemic shaped multiple levels of the developmental ecology. The negative impacts of pandemic-related child care disruptions are well-documented, highlighting increases in stress and mental health symptoms, educational setbacks, and social development (10, 20, 22). We built on this literature by examining child care service changes as they relate to children's daily lifestyles. This enabled us to obtain detailed knowledge on the interactions between changes across various developmental settings (i.e., child care and home contexts). In line with our third hypothesis, child care changes early in the pandemic were related to children's activity patterns, shown through a greater likelihood of displaying a *Screenies* or *Balanced* activity profile over the *Analog* group. This finding exemplifies the downstream effects that child care changes likely imparted on children's daily lives and that they may be proxied through children's activities. *Screenies* and *Balanced* children specifically showed higher screen use relative to other activities and compared with the *Analog* group, implying that child care disruptions may have been linked with more coping via technology. This is plausible given evidence suggesting that screen-based devices played a major role in children's social, academic, and recreational functioning during COVID-19, for better and for worse (48, 55). In contrast, the *Analog* group may represent a small subset whose lives maintained more normalcy due to avoiding child care disruptions. This may have led to lower reliance on screens and more engagement in other activities. Of note, it is possible that children in the present study maintained similar lifestyles before and after the pandemic. Ongoing longitudinal evaluations are warranted to further explore the extent to which changes in children's activities preceded or followed changes in child care utilization.

The parental sociodemographic characteristics that were linked to membership in the *Screenies* and *Balanced* groups—female gender, lower educational attainment (i.e., not having attended university)—may further highlight factors that predisposed children to experiencing higher degrees of pandemic-related disruption. The disproportionately high burdens of COVID-19 on female caregivers, largely due to higher parenting and household labor demands, are well-documented (56, 57). It is possible that female caregivers in the present study were more affected by COVID-19, resulting in spillover effects on children that were detected through activity profiles involving higher parental concerns. Based on the strong links between parent education and family socioeconomic status, having a caregiver with lower education status may also have exacerbated the impacts of pandemic-related disruptions (58). In the present study, the challenges faced by some families and caregivers may have been reflected in children's likelihood of falling into the *Screenies* and *Balanced* profiles—activity patterns associated with greater experiences of child care disruption. Interestingly, older parental age also predicted membership in these two groups, whereas some work has indicated that older caregivers were less likely to report negative family outcomes in the context of the pandemic (59). However, older caregivers may also be less likely to limit their children's screen time (60). As this activity was lowest in the *Analog* profile, older caregivers in the present study could have placed fewer restrictions on their children's screen-based activities, thereby increasing membership in the *Balanced* or *Screenies* profiles. This mechanism is speculative, and further research is required to evaluate this possibility. Ongoing work should also continue to investigate family-based factors linked with pandemic-related responses and how they translate into children's engagement with various activities to inform the supports that are best suited to each family unit.

Another study goal was to examine the associations between children's activities and caregivers' intentions to use child care services following the COVID-19 pandemic. This may generate insight into the children and families who are most in need of services as informed by children's lifestyles. We anticipated that activity profiles would be differentially associated with differences in parents' post-pandemic child care service utilization intentions. This hypothesis was partially supported: Caregivers of children in the *Balanced* profile were more likely to report planning to have their children attend services post-pandemic compared to parents of *Analog* children. Taken with the slightly higher levels of parental concern and greater likelihood of experiencing child care disruptions associated with the *Balanced* profile, this may reflect that these children reacted more strongly to COVID-19 disruptions. Their caregivers also could have felt more demands, leading to requiring more support from child care services. It is also possible that children in the *Balanced* profile required more hands-on parenting to maintain high engagement across a diverse set of activities. Again, the burdens of sustaining this level of involvement for their children may have resulted in greater inclinations to use child care. Additional research that directly explores caregivers' motivations behind child care use, including consideration of their perceptions of developmental opportunities and children's activities, is needed to substantiate these possibilities.

Regarding demographic characteristics, we found that younger caregivers were more likely to report planning to enroll children in care, which again highlights that some groups of parents were more heavily burdened and therefore in greater need of child care support during COVID-19 (61). Interestingly, caregivers who attended university showed similar patterns. Previous research suggests that those with higher education are more likely to hold careers in sectors that are more amenable to flexibility and working from home (62). It is possible that university-educated parents were more likely to face the stressful act of balancing parenting with tending to children's needs at home. Difficulties balancing remote work and parenting responsibilities during COVID-19 have been an overwhelming source of distress for many caregivers (63). In line with this, caregivers in the present study were more likely to report planning to use child care if at least one family member was working from home. Our findings may therefore reflect a need to provide families with extra support when they lack child care arrangements. Interestingly, caregivers from families in which at least one member lost their job, was laid off, or received reduced work hours due to COVID-19 expressed lower intentions to have their children attend child care when services reopen. Families who experienced employment loss may have been required to reallocate their child care expenses due to reduced financial resources. Alternatively, those who faced employment loss could have felt more well-equipped to care for their children at home due to lower occupational constraints. Further studies are required to better elucidate the motivation behind parents' decisions in relation to economic impacts on family units. Nonetheless, our results collectively highlight several key factors which may help identify families with greater needs for child care services during the pandemic. Caregivers' decisions to use services may vary based on both the sociodemographic characteristics of the family and children's activity patterns. Hence, policymakers must carefully consider the experiences of the overall family unit when making decisions about child care service availability throughout the pandemic, bearing in mind those who are most in need of support.

### 4.4. Limitations and future directions

The findings of the present study must be interpreted bearing in mind several limitations. First, the results are not generalizable to a broader population, Canadian or otherwise, due to the crowdsourced nature of the ICC-PDP sample. This issue was further exacerbated by only including a subset of participants due to missing data. The ICC-PDP dataset also included little information about the children of the caregivers who participated in the study, such as child age and gender, which may impact their time use (64). To address these limitations, future research should include a more diverse set of participants to improve generalizability.

There were also limitations regarding the measurement of children's activity patterns in the ICC-PDP study. Some variables lacked specificity. For instance, it was unclear as to what constitutes the category of the activity “developing other skills.” The list of activities included was also not exhaustive. Future work may consider employing alternative methods such as ecological momentary assessment to capture a more detailed and comprehensive view of children's activities. The parent-report nature of the present study is another limitation. Given that caregivers were required to balance a wide range of demands during the pandemic, their reports of concerns and children's activities may have been skewed by personal stressors. Moreover, previous work indicates that parents may over- or underestimate children's engagement in activities such as screen time (65). Hence, the use of different data collection methods (e.g., ecological momentary assessment, multi-informant reports of child activities) may help achieve more reliable reports of children's time use. Finally, the cross-sectional nature of the analyses prevented directional conclusions. Examinations of the trajectories and long-term relations between children's activities, parental concerns, and child care service utilization is an important next step, particularly as Canada begins to emerge from the pandemic and re-establish functions in the Early Learning and Child Care Service sector.

## 5. Conclusion

The COVID-19 pandemic had undeniable impacts on the lives of children and families, largely as a result of from disruptions to childcare services. The present study aimed to understand these effects in-depth by delineating patterns in children's activities at home during the pandemic, and their relations with parental concerns and child care service utilization. Findings highlight that children's activity engagement typically fell into one of three patterns, with slight differences in parental concerns between them. Notably, children were more likely to fall into groups for which caregivers held slightly higher levels of concern when they experienced changes in child care, illustrative disruptive impacts on the daily lives of Canadian youth. Caregivers' intentions to have their children attend child care following the pandemic also showed some associations with children's activity patterns, alongside sociodemographic characteristics, emphasizing that children's lifestyles may result in greater child care needs for some families. Overall, these findings suggest that the effects of child care disruptions were not uniform across Canadian families. Disparities can be documented through the characteristics of both children and caregivers, and “one-size-fits-all” supports will likely result in unmet needs for much of the population. As Canada begins to emerge from the pandemic and society resumes in-person functions, policymakers and service providers should work closely with parents to best understand each family's unique needs while navigating life post-pandemic. Doing so will ensure that child care programs are well-prepared for the future, therefore contributing to positive developmental outcomes for Canadian youth.

## Data availability statement

The data analyzed in this study is from a publicly available dataset provided by Statistics Canada. Information about the study and access to the dataset can be found at: https://doi.org/10.25318/45250006-eng.

## Ethics statement

Ethical review and approval was not required for the study on human participants in accordance with the local legislation and institutional requirements. The patients/participants provided their written informed consent to participate in this study.

## Author contributions

JZ led the conceptualization and design of the study, performed statistical analyses, and wrote the full draft of the manuscript, under the guidance of JS and DB. DB and JS provided assistance with analyses and feedback on the manuscript. All authors contributed to the article and approved the submitted version.
